# Shame-Sensitive Public Health

**DOI:** 10.1007/s10912-024-09877-7

**Published:** 2024-07-23

**Authors:** Fred Cooper, Luna Dolezal, Arthur Rose

**Affiliations:** 1 Wellcome Centre for Cultures and Environments of Health, University of Exeter, Exeter, UK; 2 University of Bristol Law School, University of Bristol, Bristol, UK

**Keywords:** Shame, Stigma, Public health, Covid-19

## Abstract

In this article, we argue that shaming interventions and messages during Covid-19 have drawn the relationship between public health and shame into a heightened state of contention, offering us a valuable opportunity to reconsider shame as a desired outcome of public health work, and to push back against the logics of individual responsibility and blame for illness and disease on which it sits. We begin by defining shame and demonstrating how it is conceptually and practically distinct from stigma. We then set out evidence on the consequences of shame for social and relational health outcomes and assess the past and present dimensions of shame in the context of the Covid-19 pandemic, primarily through a corpus of international news stories on the shaming of people perceived to have transgressed public health directions or advice. Following a brief note on shame (and policymaking) in a cultural context, we turn to the concept and practice of ‘shame-sensitivity’ in order to theorise a set of practical and adaptable principles that could be used to assist policymakers in short- and medium-term decision-making on urgent, tenacious, and emerging issues within public health. Finally, we consider the longer consequences of pandemic shame, making a wider case for the acknowledgement of the emotion as a key determinant of health.

In January 2021, the advertising agency MullenLowe produced a public health campaign for the UK government, titled ‘Can you look them in the eyes?’. Targeted at those ‘who were unsure or didn’t believe’ the ‘Real Risk of COVID-19’, the campaign ‘featured the people who had experienced the very worst of the pandemic—the patients and NHS staff on the frontline’ (mullenlowe.co.uk 2021). In its print and poster forms, the campaign was characterised by striking images of patients, wearing oxygen masks, staring at the camera with an accusing gaze; these images were then superimposed with variations of the tagline ‘Look her in the eyes and tell her you never bend the rules’. The visual layout worked to emphasise the two parts of the instruction by breaking the line after ‘eyes’, framing the patients’ eyes in the intervening gap. As one commentator observed, drawing on the work of Melissa Bateson, ‘images of eyes prompt a feeling of being observed, which encourages people to behave in line with social expectations’ (Magee 2021; Bateson et al. 2006).

According to surveys by the consultancy firm Kantar, the campaign was very effective. Some 87% of those polled recognised it in some form or another, while 79% of those who had not believed the risk agreed with the prompt that ‘the ads made me realise it’s vital to follow the guidelines’ (mullenlowe.co.uk 2021). But, more nuanced data collection found the moralising nature of the ads cultivated ‘highly negative responses’, including observations of ‘blaming’ and ‘guilt-tripping’, which legitimised ‘the othering of non-compliant people’ (McClaughlin et al. 2023). Where the aim of good public health messaging is often to develop social cohesion, this campaign drew on an ideology of compliance to emphasise divisions between those who obeyed and those who bent or broke the rules (Trostle 1988; Greene 2004). This, in turn, pushed some respondents to resist or dismiss the content of the ads and reject its consequences (McClaughlin et al. 2023). Even people who thought of themselves as compliant worried at the implied accusation that they had not been following the rules.

The ‘Look them in the eyes’ campaign successfully targeted an imagined group as ‘non-compliant’, and therefore worthy of naming, blaming, and shaming. This proved effective in casting as wide a net as possible over people who might identify with this group, however reluctantly. As a result, it has been criticised for creating stigma and promoting guilt (McClaughlin et al. 2023). But the campaign also, by its very imagery, poses critical questions for the role that leveraging shame—and other negative emotions—plays in public health. Although our research to date has focused on the specific context of the UK, we demonstrate here that this is by no means an isolated or parochial concern. As a powerful driver of behaviour and decision-making, shame has frequently been positioned as a useful ‘tool’ in public health practice to motivate positive behaviour and lifestyle change in line with population-level health goals (Callahan 2013). In the heightened context of the Covid-19 pandemic, there has also been outspoken support for using shame and shaming as a means to encourage people to conform to public health objectives (Pierre-Louis 2021).

The use of eyes in ‘Can you look them in the eyes?’, therefore, was a meaningful, purposive choice. Aristotle cites the Greek proverb from Euripides, ‘the eyes are the abode of shame’ (Aristotle 1994, 214–215); if shame always has a real or imagined audience, this technique conjures a pair of ‘watching eyes’ to hold the viewer accountable to their actions, finding its efficacy in the artificial generation of self-other-awareness (Bateson et al. 2006; Dear et al. 2019). However, behind the overt moralising of the campaign, which easily provokes critique, rests a series of assumptions over the prosocial benefits of shame in public health messaging, assumptions which pertain even in less dramatic and visceral examples. While public health theorists present a relative degree of consensus on the potential harms of stigma, evidence which demonstrates that shame and shaming create extensive ill effects in health and health-relevant behaviour is frequently overlooked (Dolezal and Lyons 2017). Instead, debates about shame’s use in public health have tended to focus on efficacy, rather than ethics, justice, or the risk of reputation damage or scapegoating (Lupton 2015). One qualitative study that observed a negatively inflected passivity in response to fear- and shame-inducing public health campaigns responded to this finding by advocating future research to ‘usefully deploy’ shame as a motivating emotion (Brennan & Binney 2010; Lupton 2015). Even when policymakers do not set out to use shame as an overt tactic for behaviour change, it can often be an unintended consequence of public health messaging and interventions (Dolezal and Spratt 2023). In the specific case of ‘Look them in the eyes’, this produced resistance and dismissal of the messaging (McClaughlin et al. 2023). But it also reflects a more general observation that feelings of shame produce unpredictable outcomes. A measure of this unpredictability may be found in the range of responses that Donald Nathanson tracks in his ‘shame compass’; these range from attacking others to attacking the self, from withdrawing from situations to denying their effects, signalling the multiple forms through which shame avoidance can manifest (Nathanson 1992). This unpredictability means that even a carefully designed policy can lead to secondary or inadvertent shaming in the populations being targeted, with serious consequences for uptake and engagement, and for health-seeking behaviour in the longer term (Northrop 2017).

In this article, we argue that shaming interventions and messages during Covid-19 have drawn the relationship between public health and shame into a heightened state of contention, offering us a valuable opportunity to reconsider shame as a desired outcome of public health work, and to push back against the logics of individual responsibility and blame for illness and disease on which it sits. Shame, we suggest, needs to be treated as a separate entity, as routine conflation with stigma means that many negative experiences related to public health interventions are not adequately understood or recognized. We begin, therefore, by defining shame and demonstrating how it is conceptually and practically distinct from stigma. Although the two are frequently related, it has to be acknowledged that shame can occur independently, particularly in health-relevant contexts (Dolezal 2022b). We then set out evidence on the consequences of shame for social and relational health outcomes and assess the past and present dimensions of shame in the context of the Covid-19 pandemic, primarily through a corpus of international news stories on the shaming of people perceived to have transgressed public health directions or advice. Following an exploration of shame (and policymaking) in a cultural context, we turn to the concept and practice of ‘shame-sensitivity’ in order to theorise a set of practical and adaptable principles that could be used to assist policymakers in short- and medium-term decision-making on urgent, tenacious, and emerging issues within public health (Dolezal and Gibson 2022). Finally, we consider the longer consequences of pandemic shame, making a wider case for the acknowledgement of the emotion as a key determinant of health.^1^

## Stigma and shame

There is overwhelming evidence that stigma negatively impacts the efficacy of health interventions, while also exacerbating health inequalities (Link and Phelan 2001; Stangl et al. 2019; Weiss et al. 2006). As a result, the interlocking principles that illnesses should be destigmatised (as far as this is ever fully possible), and that public health interventions should avoid creating or heightening stigma in the work that they do, are rarely directly contested (Brewis & Wutich 2019). International public health literatures generally demonstrate an informed handle on the ill effects of stigma, developed in part through visible interventions on global challenges such as obesity, mental illness, and sexually transmitted diseases (WHO 2013, 2017, 2019). Given this relative consistency in how stigma has been theorised, if not always avoided in practice, global health bodies such as the World Health Organization (WHO) and the US Centres for Disease Control (CDC) were quick to identify it as a potential problem of Covid-19, and there has been a corresponding explosion of scholarship on the topic since 2020 (Unicef/WHO/IFRC 2020; CDC 2020). This suggests that stigma remains the dominant concept used to explain and research the social burden that frequently accompanies illness. As a result, considerations of the ill effects of shame in health-related arenas are often subsumed under a concern, or conflation, with stigma (e.g. Brewis & Wutich 2019; Tyler 2020).

While living with stigma is likely to result in experiences of shame (Scambler 2004, 2018), shame and stigma should be understood as two distinct (if closely related) problems. Historically, stigmas have been marks afflicted on people who are deemed ‘inferior’, ‘deviant’, or ‘damaged’ in some way, in order to render them visible and identifiable to a community. Stigma is a social or political phenomenon, where individuals are unfairly labelled as ‘not worthy’ or ‘not good enough’ in a dominant social order. Stigma is not something we experience directly, in the same way as we experience pain or fear. Instead, when stigmatised, we may experience a range of other negative phenomena, such as discrimination, unfair treatment, labelling, and stereotyping, which become inscribed on our minds and bodies through affective experiences such as shame, anxiety, stress, and worry (Dolezal 2021). In short, stigma is a label or category, while shame is an affect or emotion. While shame is not always caused by stigma, it is clear that stigma structurally contains the possibility of shame or shaming, where individuals may experience or anticipate negative judgement from others because of their stigma. More frequently, however, scholarship on stigma which addresses shame combines category slippage with misleading causative assumptions, overlooking the significant point that not all shame is grounded in stigma.

Indeed, this important nuance has been particularly evident in how shame has been present in the Covid-19 pandemic. In many countries, the wearing of compulsory face masks and guidelines for physical distancing in public spaces have been conspicuous public health responses; in different times and places, however, both ‘compliance’ and ‘non-compliance’ with these measures have been subject to shame and shaming, highlighting the complicated negotiations between health professionals, political authorities and publics behind the reification of certain medical recommendations over others (Trostle 1988; Greene 2004). When masking has been mandated by law or strongly advised and widely participated in, refusal or forgetfulness has been a source of judgement, social evasion, and sometimes outright shaming; this has likewise been the case for keeping a distance deemed appropriate to make viral transmission less likely (Hess 2020). Conversely, in phases of the pandemic where mask-wearing was yet to be widely adopted, or had been largely abandoned, wearing one has been construed as a signifier of illness or a marker of undue caution (Capraro and Barcelo 2020). The testimony of a Syrian refugee in Sweden, Habib, offers a useful understanding of how decisions around mask-wearing were conditioned by government advice, the threat of familial shame, and broader systems of knowledge about their global use:

My oldest child [16] says that the authorities say that we should not wear masks, that masks don’t protect against corona. Here you never see people in masks, not in the shops, not on the train. But all over the world you see people with masks, and they have rules and have to wear them. I thought we should buy masks but my daughter said no. She said that she would be ashamed if I walked around with a mask. But, if the government in other countries say you should have a mask, why is it different here? It is the same virus. (Wissö and Bäck-Wiklund 2021, 7)

Similarly, when keeping a pronounced physical distance is considered unnecessary by the majority of people in a public space, those who continue to do so are rendered hypervisible by the contextual absurdity of their attempts at avoidance. Although there is plenty of room in these examples for shame and stigma to interrelate—particularly through mediators of risk from Covid-19 which can make protective behaviours more likely, such as disability or racialisation—it does not follow that they will necessarily do so (Hearne and Niño 2022). While pandemic shaming has always been inflected with pre-existing systems of stigmatisation, it has also frequently transcended them; often encouraged in public health rhetoric, a heightened surveillance of individual behaviour has accompanied the imposition of new norms and boundaries to transgress (Cooper et al. 2023).

But what is shame—as distinct from stigma—and why does it matter? Shame, we argue, is best defined as a negative, self-conscious emotion. Shame occurs when people feel themselves to have been seen and judged to be flawed in some crucial way, with some aspect of their identity or selfhood perceived to be inadequate, damaged, inappropriate, or immoral. While it can sometimes be related to guilt, guilt becomes shame when the worth of the person—as opposed to a negative valuation of isolated behaviour—is thought to be at stake, often through the social and cultural magnitude of the guilt-inducing act (Miceli and Castelfranchi 2018). While shame can stem from the personal transgression of laws, rules, and values, it is also a political emotion, a matter of how individuals are positioned in relation to unjust structural circumstances or social norms. It can be usefully understood, therefore, as the lived and subjective emotional, psychological, and physiological correlate or response to situational or systemic judgement, degradation, or exclusion. Variants of shame include a wide array of negative self-conscious experiences such as embarrassment, humiliation, chagrin, mortification, feelings of defectiveness, heightened self-consciousness, and low self-worth (Retzinger 1995).

Shame itself can also be a potent source of shame; admitting to the experience can be difficult, and shame is frequently concealed or avoided as a result. Precisely because it is often hidden and unspoken, shame is a powerful force in personal experience and interpersonal encounters. People go out of their way to avoid shame, even when patterns of avoidance are self-defeating or destructive; for many, escaping shame can feel like a life-saving measure (Marcinko, Bilic and Eterovic 2017). Experiences of shame can also be heightened for certain populations. For instance, feminist scholars have long noted the structural shaming experienced by women and others who are minoritised, discriminated against, or positioned lower down a social hierarchy (Bartky 1990; Dolezal 2015; Fischer 2018, Harris-Perry 2011). Those who live with stigmatised identities, circumstances, or attributes, for instance, addiction, homelessness, minority status, and experiences of poverty, lack of literacy, obesity, chronic illness, loneliness, or disability, may live with experiences of ‘stigmatising shaming’ (Harris-Perry 2011). Chronic shame, resulting from adverse experiences such as trauma, prolonged discrimination, or other social harms, can lead to avoidance behaviours such as substance abuse, social withdrawal, self-harm, and suicide (Dolezal 2022a). Chronic or persistent experiences of shame also cause prolonged stress in the body, with a clear physiological effect on the immune and cardiovascular systems. This can lead to or exacerbate ill health, through the chronic elevation of cortisol levels (Lewis and Ramsay 2002; Dickerson et al. 2004).

For these reasons, shame is pivotal to a good understanding of how ill health is actively lived, and how people interact with healthcare (and other) services (Dolezal and Gibson 2022). Shame and embarrassment are common experiences for patients in healthcare settings, and this frames how clinical encounters are imagined and anticipated; (prospective) patients often fear being judged and/or shamed by health professionals, particularly as such encounters are frequently accompanied by the exposure of their vulnerabilities and physical bodies, along with their (perceived) flaws, inadequacies, faults, or frailties (Dolezal 2015). Experiencing or anticipating shame can add to the burden of illness in a variety of ways. Shame can lead to avoidance or procrastination in seeking medical attention, even when serious symptoms are experienced. It can lead to the concealment of a diagnosis from family or friends, or the failure to disclose important details of health status, situation, or identity in a clinical encounter. It can lead to the avoidance of testing for infectious illnesses (such as HIV, Hepatitis C, or Covid-19), as well as failure to take up or complete courses of treatment (Dolezal and Lyons 2017). As this article asserts, it also colours how people respond to public health messaging, initiatives, and advice. Even when the witting or unwitting use of shame in public health appears to be successful, in terms of leveraging short-term behavioural changes, the negative repercussions can be unpredictable and extensive.

## Shame and Covid-19: A global challenge

The Covid-19 pandemic has been the context for widespread experiences of shame and shaming, particularly in regard to groups or individuals perceived to be transmitting the virus, breaking social distancing guidelines, or ignoring public health directives. These have included—but by no means been limited to—healthcare workers, people unable to wear face masks, the vaccine-hesitant, young people making use of public spaces, and those—such as commuters or Black Lives Matter protestors—for whom economic, social, religious, political, cultural, or relational motivations outweigh or compete with public health imperatives. Covid-19 has also worsened the experiences of populations already subject to persistent shaming, whether through racialized concerns over ‘contamination’ and global mobility, or stigmatising narratives on Covid-19 and overweight bodies (Cooper et al. 2023). Frequently, experiences of shaming have been intersectional, cutting deepest where people and groups with long experiences of being publicly shamed became tangled in newer dynamics of viral shaming (Mayer and Vanderheiden 2021). To a degree, many of these problems could plausibly have been anticipated. Shame and disease have a well-evidenced and documented relationship, and there are relevant literatures, particularly on HIV/AIDS, which could have informed valuable learning on shame and Covid-19 (Arnold 2021). In failing to sufficiently predict and address shame, many public health systems have limited the effectiveness of their own responses to Covid-19; allowed shame to further increase the social and relational burden of the pandemic; and made unnecessary and harmful room for explicit instances of shaming, through or in service to public health objectives.

Globally, non-compliance with public health measures has been the justification for numerous scenes of shame and shaming. In part, this reflects the (at times awkward) co-option of different groups and agencies in doing or supporting public health work, such as police officers and elected officials. Towards the beginning of the pandemic, videos of Italian mayors condemning the behaviour of citizens gained international attention, with ‘little public consideration for whether such stigmatization… [was] proportionate or effective’; in the Netherlands, calls for Covid-19 patients with bad prognoses to make space in hospitals for those with better chances of survival shifted blame for poor outcomes onto those who refused (Pelizza 2020). In China, India, and Indonesia, police have made rule-breakers submit to public shaming, forcing them to stand or parade in public with placards detailing personal information or promises to comply in the future, and uploading pictures of offenders to social media (BBC 2021; Bagcchi 2020; AFP 2020). In the UK and Canada, broader political discourses on individual irresponsibility have been accompanied by shaming news coverage on ‘covidiots’, drawing attention to the seemingly blameworthy actions of people unable to justify themselves to officials. In the UK at least, this was part of a far wider culture of shaming encouraged and enlivened by political and public health rhetoric (Capurro et al. 2022; Cooper et al. 2023). Additional research in the UK context has taken sight of public health messaging around obesity, arguing that attempts to raise awareness about the correlation between excess weight and Covid-19 morbidity and mortality have placed an unnecessary burden of shame on the people they target. Messaging on losing weight to ease the burden on health services misrepresents complicated challenges around exercise and healthy eating as matters of simple choice, implicating individuals in entrenched systemic problems beyond their capacity to influence (Dolezal and Spratt 2023; Le Brocq et al. 2020).

Shame has also gathered—or been assembled—around healthcare workers, keying into anxieties over increased risk of contamination, a legacy of the early stages of the pandemic where the majority of cases were assumed to be present in hospitals, rather than communities and workplaces. Canada, Australia, and Poland each had particularly visible instances of doctors being shamed for supposedly spreading Covid-19, in some cases with disastrous consequences (Dolezal 2021; Cooper et al. 2023). Healthcare professionals have also been subject to shaming in Mexico and Malawi, where they were denied access to public transport and ostracised by neighbours in India (Bagcchi 2020). In Croatia and the UK, they have been further shamed for exhaustion and burnout, particularly in the context of inflated and damaging ideals of national sacrifice (Marčinko et al. 2021; Cooper et al. 2023; Kohlt 2020).

Predictably, catching or spreading the virus has also been a significant source of shame (Peters et al. 2022). Qualitative work with Israeli Covid-19 survivors has traced the nuances of shame at different stages of the disease, from the moment of discovery through to illness, infectiousness, potential hospitalisation, and convalescence. Participants stressed their desire to conceal the diagnosis, the guilt and shame of putting others at risk, the humiliation and exposure of medical treatment, and, in one passage that bears repeating, the abandonment and shame of physical quarantine: ‘They treat you as if you were a leper. I felt completely alone. That I am repulsive. As if anybody who would touch me would also become a leper’ (Dopelt et al. 2023). Research into the shame experiences of respondents living in Germany, Italy, South Africa, the US, Portugal, Canada, Germany, Australia, the Netherlands, the UK, Nigeria, and India has also addressed similar themes, articulating the significant emotional and relational burden that shame over Covid-19 has imposed (Mayer and Vanderheiden 2021). Reflecting the widespread ambivalence over shame in public health that the present article addresses, Mayer and Vanderheiden also note that the emotion can have useful effects, such as increased mask-wearing or compliance with public health regulations; in this vein, they reproduce the testimonies of participants who reference ‘the positive effect of shame as a mechanism of social control’ (Mayer and Vanderheiden 2021, 9). This risks recapitulating precisely the uncritical acceptance of compliance as an unalloyed good, whose pursuit justifies any potential collateral damage (Trostle 1988).

These examples make it clear that shame has been a problem of (and for) public health work in a number of different cultural contexts. While some variant of shame is felt in almost every human society, the ways in which shame is experienced and responded to—as well as the spectrum of actions, feelings, experiences, and behaviours that are considered to be shameful—are conditioned by specific personal, social, political, economic, cultural, and historical processes and circumstances. Moving beyond outdated and simplistic theorisations of nation-level ‘shame cultures’, recent research on shame and culture calls for a closer attentiveness to how shame is produced and experienced in real-world relationships and situations (Cozens 2018). Broad cultural ideologies and practices around shame can help to frame and situate it as a lived experience, but culture has to be understood as complex, local, and in a perpetual state of redefinition and reproduction. The landmark WHO report "Culture Matters" highlights UNESCO’s useful definition of culture as comprising ‘lifestyles, ways of living together, value systems, traditions and beliefs’, with national, religious, or ethnic affiliations as only part of the picture (Napier et al. 2017, x).

Research on shame across cultures further emphasises that the English word ‘shame’ does not translate cleanly into other languages, with proximal words for shame carrying diverse weights and meanings which frequently escape outside observers. Work—such as the present article—which speaks to the problem of shame in multiple cultural contexts can only offer a loose overview of shared public health challenges. The ways that shame is thought, spoken, and felt will always differ by cultural context, if only fractionally. As Karolina Krawczak argues, drawing on the work of the linguist Anna Wierzbicka, it is ‘important that we avoid “absolutizing” the concept [shame] as “a universal human emotion” on the basis of its meaning in a given language, such as English’ (Krawczak 2014, 443; Wierzbicka 1999). This is not to suggest that the experience of shame is entirely contingent on cultural, social, and linguistic conditions; rather, that universal claims about shame, routed through specific languages, fail to appreciate the nuances of particular cultural triggers. There may be a case for thinking of shame, at least in some respects, as a human constant, but all human constants are nevertheless culturally and historically bounded, as work on pain has shown (Bourke 2014). In practice, this means that shame, in terms of how it is produced and experienced, can be different for different people in different times and places. For example, tracking the connotations of shame and its proximal words across a series of cultures on the individualism-collectivism axis, Krawczak suggests that more collectivist cultures, ‘characterized by a relatively higher degree of interdependence and lower interpersonal mobility’, produce experiences of shame which are more closely defined by the threat posed by shameful acts to interpersonal relationships and community standing (Krawczak 2017, 19).

Although the nation is frequently the standard unit of measurement for scholarship on the cultural contexts of health, it should of course be acknowledged that each country encompasses multiple porous and shifting cultures with different relationships to shame (Kollareth et al. 2018). In Canada, the Maritime provinces—New Brunswick, Nova Scotia, and Prince Edward Island—have been identified as particular sites for increased community surveillance and public shaming, in part because of early successes in containing the virus, but also because of the presence of smaller, tight-knit communities (Fleguel 2021). In the UK, a government decision to keep the city of Leicester in quarantine at a point where restrictions elsewhere were easing was experienced by residents as directly increasing their burden of shame, creating a local context where shame had a significantly different valence than in neighbouring cities such as Nottingham or Birmingham (Dolezal and Spratt 2023). What might be shameful in one context, culture, or environment may not be in another, and the ways and sites in which shame is experienced will also differ. Bodily shame, for example, differs extensively according to the kinds of bodies that are valued or devalued in any given context. Literatures on shame which originate in countries benefiting from extensive existing research, including the present article, can only take public health policymakers in other contexts so far. Attempts to prevent or alleviate shame should always be attentive to cultural contexts and national and local histories and systems of knowledge (Roberts 2001, 123).

## Shame-sensitive public health

Layered through the differences in context noted above, each cultural framing of shame has been shaped and produced, at least in part, by unique histories of shame as a behavioural tool in political rhetoric and public health messaging (Cooper et al. 2023). These histories contribute to differences in how shame around health and illness is felt, but they also create institutional cultures which can either support or obstruct compensatory measures, such as the development and application of shame-sensitive practice. In healthcare, shame has been identified as an ‘affective determinant of health’, and there is clear evidence that shame and strategies for shame avoidance both exacerbate and cause negative health outcomes (Dolezal and Lyons 2017). While shame and shaming can sometimes motivate positive behaviour and compliance with public health guidance, the effects of shame are unpredictable. As evidenced by Nathanson’s ‘shame compass’, we can never guarantee how shame will ‘land’ on an individual and the behaviours or reactions that it will provoke. The volatility of shame, coupled with its potential to lead to negative social and health outcomes, means that it is not a dependable ‘tool’ for public health practitioners. Shame is not something that can be reliably deployed across a population, and its intentional use should always be avoided as there is no guarantee it will not result in harm, rather than good.

As a result, it is imperative that healthcare professionals and organisations become ‘shame-sensitive’ (Dolezal and Gibson 2022). Shame-sensitivity acknowledges that shame is inevitable and that interactions with health services, or other professional bodies, can invoke or exacerbate shame, especially as a result of vulnerability, unequal power relations, and structural inequalities. In broad terms, adopting shame-sensitivity in healthcare entails being alert to the effects of shame, being able to identify, and mitigate against, shaming policies and practice, and being able to sensitively manage shame and shame dynamics in clinical encounters and within professional practice (Dolezal and Gibson 2022).

As discussed above, shame is easily incited by public health initiatives, policies, and practices. We therefore suggest four brief recommendations for shame-sensitive public health. Intended to be neither definitive, didactic, nor universal, these recommendations are offered as a starting point for policymakers interested in adopting evidence-based principles on avoiding or mitigating shame in their own practice and the institutions they work within.

1. Reject shame and shaming as behavioural tools in policymaking or practice. Not all shaming is accidental, and many initiatives and encounters still rely on shame as the inherent emotional driver of the change they set out to promote. An institutional commitment to eliminating explicit shaming in policy and practice, as well as continually interrogating policies and practices for inadvertent shaming, is an effective starting point.
2. Build attentiveness to shame into institutional expertise and cultures, through the development of ‘shame competence’ (Dolezal and Gibson 2022), along with shared tools and resources. Shame competence involves a systematic, nuanced, and collaborative understanding of how shame is produced and experienced. Through a foundation of shame competence among individual practitioners and within organisations, mutually agreed goals and frames of reference can be developed; this could take the form of an institutional code of conduct, or a shame-proofing toolkit.
3. Use these tools and competencies to conduct frequent and challenging reviews and audits on work of any description which has the potential to generate, spread, or exacerbate shame. Likewise, apply shame competence to pre-existing and emerging public health problems, asking whether, when, where, how, and for whom shame might be present, or likely to arise. Shame-sensitivity in public health entails both a commitment to avoid representing people, choices, or behaviour in ways which could cause shame, and sustained critical reflection on how existing feelings of shame can be minimised and mitigated.
4. Engage and collaborate with excluded communities and publics to promote shame-conscious health-seeking or risk-averse behaviour, and support them proactively to do so, including by fostering supportive networks and relationship-based practice. Shifting emphasis away from individual decision-making—and understanding that this approach creates shame—makes space for attention to the collective determinants of health, trust, belonging, dignity, and equity.

What these principles do is open up an alternative way of thinking about the emotions and how campaigns, actions, images, and rhetoric in the service of public health activate or exploit them. Had shame-sensitivity been significantly embedded in the processes and institutions that create public health work during the pandemic, it is reasonable to suggest that such work might have taken place in a far less harmful and divisive affective register. For the reasons we outline above, this would have lessened the burden of shame in two ways; by avoiding unnecessary and artificially imposed instances of shaming, and by structuring a heightened attentiveness to shame where it otherwise occurred.

## Shame and public health ‘after’ Covid-19

Pandemics rarely—if ever—end cleanly (Greene and Vargha 2020). Prefixing Covid-19 with ‘post’, even in speculative or future-facing work, is an uncomfortable act of writing. The assertion that we are ‘post’ something—represented in this case by a supposed shift to endemicity—can be profoundly politically loaded, putting the illusion of historical distance between ongoing challenges and our ethical responsibility to address them (Valluvan 2016). Indeed, pandemic temporalities are not collectively or evenly experienced, but fragmented and fractured; for many, the virus is just as pressing a risk—and a constraint on everyday life—as it was in 2020 or 2021. The bereaved, traumatised, acutely or chronically shamed, post-virally disabled, and otherwise significantly harmed might plausibly be described as being ‘post-covid’, but in the sense that many countries in the late 1940s, 1950s, and 1960s were ‘post-war’, still living the reverberations of a shattering and seismic rupture, an era-defining catastrophe with a long and violent shadow.

What scholarship and activism on the pandemic has done—and continues to do—is bring a raft of health, economic, social, and political problems into more visible states of contention (Scambler 2020a). It may not be possible to think of Covid-19 as ‘over’ for a very long time, but it has been repeatedly superseded in political and public imaginations by new crises and emergencies. The question, therefore, becomes about how to best mobilise the systems of knowledge that have been created around—or deepened by—Covid-19, whether in response to the present pandemic, as a new lens on the historical challenges and inequalities that framed how it landed, or as a form of preparedness for emerging diseases and disasters (Engebretsen and Baker 2023).

In this context, our research identifies a significant and pressing need for public health systems to grapple meaningfully with the problem of shame. This omission pre-dated Covid-19, though, as we have shown, it had particularly extensive and damaging consequences for public health responses to the virus. At worst, interventions and communications which actively relied on shame—or which promoted it unwittingly through inattention to how the emotion works—have contributed considerably to the social and medical burden of the pandemic. At best, public health responses which might have been otherwise benign have been weakened by an inability to anticipate, reckon with, and manage shame in the populations they attempt to engage. One important lesson that can be extracted from Covid-19 is that policymakers urgently require good evidence on shame, how it works, and its short- and long-term harms, and effective scaffolding to transition from interventions and messages which rely on or make room for shame to interventions which identify, acknowledge, and work proactively against it.

Alongside this necessity, which can be considered a vital component of future pandemic and crisis preparedness, sits a pressing need to resolve the social, relational, and medical legacies of heightened shame over the past 4 years. For many, shame over viral transmission or poor pandemic citizenship might have been something painfully new; for others, prior encounters with shame framed and conditioned the ways that pandemic shame was experienced and felt. In both cases, the deep or shallow marks left by shame work against vital determinants of health. They compromise positive and protective feelings of relational embeddedness and stoke mistrust in social and medical systems. They contribute to feelings of isolation and alienation, whether incremental or acute; in extreme cases, they can ignite a lifelong relationship with shame which severely curtails the possibility for security, connection, or social and political engagement (Dolezal 2022a). In being shamed, whether by politicians, public health initiatives, or other members of the public, individuals and populations can become shame-prone, more sensitive to future instances of shame, and less likely to engage fully in health systems or health-seeking behaviour. Shaming easily misfires, especially when targeted at vulnerable or shame-prone populations. Rather than being a pro-social force that motivates positive behaviour change, shame can easily lead to defensiveness, disengagement, and disempowerment (Nussbaum 2004). In attaching most forcefully to groups who have long experiences of being lower down social hierarchies, pandemic shame should also be considered as a significant vector for, and a component of, entrenched health inequalities (Scambler 2020b).

Already undergoing painful processes of collective trauma and grief, populations subjected to shame are populations with weakened capacities to stay well. Although this context for future policymaking may seem daunting, the long-term effects of shame are neither inevitable nor irreversible. We renew, therefore, earlier calls for shame to be acknowledged as a vital determinant of health (Dolezal and Lyons 2017). Shame is an indispensable theoretical tool for understanding public behaviour in the pandemic, the processes by which health inequalities result in uneven viral outcomes, and the ways that adverse social, emotional, and relational experiences can threaten collective health in the longer term. Careful attention to shame in future public health work can help ward against rehearsing the same patterns of shaming as a means of behavioural inducement and begin to address some of the complex legacies of the emotion—including during Covid-19—for shamed individuals and communities.

## Data Availability

No new data was generated for this research.

## Declarations

1
**Endnotes**
This article draws on—and significantly expands and diversifies—a short briefing note written for (Cooper et al. 2022) in 2022.
